# Intraneuronal accumulation of amyloid-β peptides as the pathomechanism linking autism and its co-morbidities: epilepsy and self-injurious behavior — the hypothesis

**DOI:** 10.3389/fnmol.2023.1160967

**Published:** 2023-05-26

**Authors:** Janusz Frackowiak, Bozena Mazur-Kolecka

**Affiliations:** Department of Developmental Neurobiology, New York State Institute for Basic Research in Developmental Disabilities, Staten Island, NY, United States

**Keywords:** autism spectrum disorder, epilepsy, self-injurious behavior, amyloid-beta peptides, amyloid-beta precursor protein, oxidative stress, transcription, cell receptors’ functions

## Abstract

Autism spectrum disorder (ASD) is associated with enhanced processing of amyloid-β precursor protein (APP) by secretase-α, higher blood levels of sAPPα and intraneuronal accumulation of N-terminally truncated Aβ peptides in the brain cortex — mainly in the GABAergic neurons expressing parvalbumin — and subcortical structures. Brain Aβ accumulation has been also described in epilepsy—the frequent ASD co-morbidity. Furthermore, Aβ peptides have been shown to induce electroconvulsive episodes. Enhanced production and altered processing of APP, as well as accumulation of Aβ in the brain are also frequent consequences of traumatic brain injuries which result from self-injurious behaviors, another ASD co-morbidity. We discuss distinct consequences of accumulation of Aβ in the neurons and synapses depending on the Aβ species, their posttranslational modifications, concentration, level of aggregation and oligomerization, as well as brain structures, cell types and subcellular structures where it occurs. The biological effects of Aβ species which are discussed in the context of the pathomechanisms of ASD, epilepsy, and self-injurious behavior include modulation of transcription—both activation and repression; induction of oxidative stress; activation and alteration of membrane receptors’ signaling; formation of calcium channels causing hyper-activation of neurons; reduction of GABAergic signaling — all of which lead to disruption of functions of synapses and neuronal networks. We conclude that ASD, epilepsy, and self-injurious behaviors all contribute to the enhanced production and accumulation of Aβ peptides which in turn cause and enhance dysfunctions of the neuronal networks that manifest as autism clinical symptoms, epilepsy, and self-injurious behaviors.

## Introduction

Amyloid-β precursor protein (APP) is an evolutionary conserved and widely expressed large transmembrane protein of multiple functions which include stabilization of neuronal calcium fluxes, cell–cell or cell-matrix adhesion and inhibition of the clotting cascade. The complex processing of APP by specific secretases leads to production of multiple fragments including Aβ peptides, mainly the 42 and 40 amino-acid long species (De Strooper et al., 2010), whose biological functions are still being characterized (Kummer and Heneka, 2014). Abundant Aβ peptides detected in CSF of infants and children—the control group in the Down syndrome study (Mehta et al., 2007) suggests their role in normal brain development. The role of Aβ in brain development is further indicated by formation of oligomeric Aβ in the developing retina, which led authors to hypothesize that Aβ oligomers may regulate neural development in the embryonal phase, functioning as a peptide hormone (Bartley et al., 2022). Excessive accumulation of Aβ — intra-or extra-cellular — in the brain can result from altered APP production, processing, clearance, post-translational modifications or aggregation, which leads to various brain dysfunctions (Hillen, 2019; Hampel et al., 2021). Massive deposition of Aβ peptides in the form of amyloid fibrils and oligomers in the brain is the well-known and characteristic neuropathological feature in Alzheimer’s disease and Down syndrome, however, accumulation of Aβ peptides in the central nervous system has also been reported in aging without dementia (Wegiel et al., 2007) as well as in several pathological conditions such as autism (Wegiel et al., 2012a,b), Fragile X (Westmark et al., 2016), epilepsy (Joutsa et al., 2017; von Rüden et al., 2020), and traumatic brain injury (Itoh et al., 2009; Johnson et al., 2010; Washington et al., 2014). Aβ detected in young individuals — controls and with autism — is different from Alzheimer’s disease: importantly it does not form extracellular deposits in the form of classical or diffused plaques composed of fibrillar amyloid.

Ethiopathogenesis of autism spectrum disorder (ASD) involves an array of genetic, epigenetic and environmental factors and is associated with dysregulation of some basic cellular homeostatic mechanisms, including processing of APP. Plasma levels of secreted APP were reported to be two or more times higher in children with severe autism and aggressive behavior than in children without autism and up to four times higher than in children with mild autism (Sokol et al., 2006; Ray et al., 2011). Secreted APP-α levels in plasma were found significantly increased in 60% of autistic children, as compared to aged-matched controls (Bailey et al., 2008), particularly in the regressive form of ASD (Li et al., 2022). The increased secretase-α processing of APP and resulting higher levels of the neurotrophic and myelinotrophic soluble sAPPα have been proposed to contribute to development of autism symptoms (Ray et al., 2011). Accumulation of N-terminally truncated Aβ (N-tr-Aβ) peptides was detected in the brain in idiopathic autism and in the duplication chromosome 15q11.2-q13 autism [dup(15)] (Wegiel et al., 2012a,b; Frackowiak et al., 2013). We hypothesized that this excessive accumulation of Aβ in the brain in autism may cause dysfunctions of neurons and neuronal networks (Frackowiak et al., 2013, 2020).

Autism spectrum disorder is associated with several co-morbidities most frequent of which are epilepsy and self-injurious behavior, frequently resulting in head trauma. We hypothesize that altered APP processing and accumulation of Aβ detected in epilepsy and traumatic brain injury are the major pathophysiological mechanisms that link autism with some of its co-morbidities.

## Autism: structures and cells involved in Aβ accumulation

Accumulation of amino-terminally truncated amyloid-β peptide (N-tr-Aβ)— corresponding to the product of secretase-α and secretase-γ, has been detected in neurons, astrocytes, microglia and neuropil in the brain cortex, cerebellum, and subcortical nuclei in children with ASD as young as 5 and 8 years of age (Wegiel et al., 2012a,b; Frackowiak et al., 2013, 2020). The N-tr-Aβ species with C-terminal 40-Val and 42-Ala were identified, as well as the peptides with N-terminal 11-Glu modified to pyroglutamate. The accumulated Aβ peptides have been characterized by immunohistochemical methods and confocal microscopy using a panel of monoclonal and affinity purified antibodies specific for several distinct epitopes and by immunoblotting (Wegiel et al., 2012a,b; Frackowiak et al., 2013, 2020). Detection of diffuse amyloid plaques in the cortex of subjects with ASD aged 39 to 52 years containing Aβ species 1–40 and 1–42 suggests activation of the amyloidogenic pathway of APP processing with β- and γ-secretases, and synaptic secretion of full length Aβ1–40/42, later in life—in the fourth and fifth decade (Wegiel et al., 2012a,b). The two alterations of APP processing may be closely linked, as Aβ17–42 was proposed to form nucleation centers and to accelerate plaque formation by deposition of more abundant full-length Aβ — the Aβ1–40 and Aβ1–42 species (Pike et al., 1995). The shorter forms may also initiate formation of oligomers detected in autism (Frackowiak et al., 2020) a fraction of which may be also hetero-oligomers.

Accumulation of Aβ peptides was detected in idiopathic autism, as well as in autism associated with a determined specific genetic alteration—maternally inherited supernumerary isodicentric chromosome 15—[dup(15)]. The latter mutation in majority of probands leads to autistic disorder and mental retardation of various levels. The clinical symptoms include autism with speech delay, lack of social reciprocity, and stereotyped behaviors, as well as muscle hypotonia, seizures, and delayed gross motor development (Wolpert et al., 2000; review Lusk et al., 2016). The thorough clinical examination allowed to exclude such patients from the idiopathic autism cohort.

Intracellular Aβ load is structure specific, affects neurons and glia and highly varies among cells—from none to high. The structures particularly rich in neurons with Aβ accumulation in dup(15) autism are the amygdala and thalamus (46% of Aβ-immunoreactive cells), significantly more than in idiopathic autism, while the control group contained low proportion of neurons with N-tr-Aβ. The intraneuronal Aβ load is also significantly greater in the autism dup(15) group than in controls and idiopathic autism in the frontal, temporal and occipital cortex, hippocampus CA1 and CA4 sectors and dentate nucleus (Wegiel et al., 2012a,b). The fraction of neurons with N-tr-Aβ as high as 60% was found among large pyramidal neurons in the frontal cortex in the dup(15)/autistic subjects; 30–45% of neurons in idiopathic autism, with a strong reaction in 3–10% cells, while control brains contained a significantly weaker immunoreactivity affecting only 15–35% of neurons (Wegiel et al., 2012a,b; Frackowiak et al., 2013). This distribution of Aβ accumulation suggests differences of regulatory mechanisms in different brain structures and neuronal populations.

In the prefrontal cortex there are two neuronal populations: glutamatergic excitatory neurons and GABAergic inhibitory interneurons, both of which contain cells of heterogenous morphology and functions. The neurons with abundant intracellular Aβ peptides in this brain region in autism have been found to be mainly GABAergic, along with a minor fraction of glutamatergic neurons (Frackowiak et al., 2020). Among the GABAergic interneurons in neocortex the most numerous subpopulations are the cells that express the Ca2^+^-binding protein parvalbumin (PVA), the neuropeptide somatostatin (SST), and the ionotropic serotonin receptor 5HT3a. These subpopulations have different embryological origins and distinct functional properties. The most frequent PVA expressing interneurons which include basket cells and chandelier cells, represent about 40–50% of GABAergic neurons, while the cells expressing SST and 5HT3a receptors represent about 30% and 20–30% of GABAergic neurons, respectively. These groups are also heterogeneous and include neurons that express the neuropeptide VIP, and other neuropeptides, which probably are involved in shaping cortical circuits during specific behavioral tasks and contexts (reviewed in Kawaguchi and Kubota, 1997; Rudy et al., 2011; Kelsom and Lu, 2013). In autism the PVA^+^ GABAergic neurons in the prefrontal cortex have been also identified as the subpopulation involved in abundant accumulation of Aβ peptides with and without pyroglutamate-11 modified N-terminus (Frackowiak et al., 2020). In autism the density of the PVA^+^ neurons in the prefrontal cortex, but not the interneurons expressing calbindin or calretinin, was reported to be significantly reduced as compared to control subjects (Hashemi et al., 2017). Decrease of this neuron subpopulation could result from modulation of neurodevelopment or oxidative damage of neurons by the accumulated Aβ.

Autism spectrum disorder cases caused by a mutation of a single specific gene make a small fraction of the whole ASD cohort. Fragile X syndrome is the most common cause of ASD linked to a single gene—in the full mutation group, 67% males and 23% females show ASD symptoms (Clifford et al., 2007). Fragile X syndrome has also been linked to higher levels of APP and Aβ in the blood and in the brain, even though the cellular localization of Aβ has not been identified (Westmark et al., 2016). Fragile X mental retardation protein (FMRP) is the protein which binds target mRNAs, regulating their transport, stability, and translation. Deficiency of FMRP alters translation leading to upregulation of APP, however, it also deregulates receptor-mediated signaling pathways and causes synaptic dysfunctions (Westmark et al., 2016), thus the input of APP and Aβ in the autistic symptoms in this syndrome is unclear.

### Subcellular localization and pathomechanisms of Aβ accumulation

The Aβ-immunoreactive granular material was detected in idiopathic and dup(15) autism in the cytoplasm and nucleus of neurons, in synapses, glia and the neuropil (Wegiel et al., 2012a,b; Frackowiak et al., 2013, 2020). In the perikaryon of cortical neurons Aβ was mainly found in lysosomes with only minor fractions in rab5-positive endocytic vesicles and LC3B-positive autophagic vacuoles and lipofuscin (Wegiel et al., 2012a,b), which indicates accumulation in organelles involved in transport, proteolysis, and storage of metabolic residues. Thus, alterations in these pathways of APP and Aβ processing and transport are the likely mechanisms of Aβ accumulation in this neuronal population. The nuclei contain a significant fraction of neuronal N-tr-Aβ immunoreactive granules —between 14 and 20% of the total neuronal load—both the unmodified and pyroglutamate modified at glutamate-11 species (Frackowiak et al., 2020). N-tr-Aβ is also accumulated in a significant fraction of GABAergic synapses—7.5 and 7.9% in idiopathic and dup(15) autism, respectively — significantly more than in controls (Frackowiak et al., 2020).

The majority of Aβ peptides that accumulate in the brain in autism are N-terminally truncated, and form SDS stable oligomers (Frackowiak et al., 2020).

The mechanisms responsible for the observed accumulation of N-tr-Aβ may include enhanced secretase-α processing of APP in ASD (Sokol et al., 2006; Bailey et al., 2008; Ray et al., 2011), as well as a decreased peptide clearance that involves transport through the perivascular drainage system and local enzymatic degradation, particularly by the Insulin-degrading enzyme (IDE), endothelin-converting enzymes (ECE)-1 and ECE-2, and neprilysin (Pacheco-Quinto et al., 2016). The two latter enzymes are mainly expressed in GABAergic neurons: ECE-2 primarily in SST^+^ neurons and synaptosomes, and neprilysin — mostly in synapses of the PVA-expressing interneurons. Synapses of GABAergic neurons were suggested to be the sites of Aβ degradation (Pacheco-Quinto et al., 2016), hence, accumulation of N-tr-Aβ mainly in the PVA^+^ but not the SST^+^ subpopulation suggests that a decreased expression of neprilysin in the former subpopulation might be involved in accumulation of N-tr-Aβ. Neprilysin has been postulated an important protective factor for neurons and neuronal progenitor cells against cytopathic effects of Aβ (Oh et al., 2016). Aβ clearance may be further reduced by aggregation and oligomerization of Aβ as well as by complexing with other proteins. Aβ and N-tr-Aβ accumulated in synapses may be further transferred to postsynaptic neurons, including glutamatergic (Domert et al., 2014) and seeding of Aβ accumulation in the latter neurons.

Accumulation of Aβ peptides may be further aggravated by increased APP and Aβ production associated with incidents of epilepsy and head trauma associated with self-injurious behavior— discussed below.

## Epilepsy: Aβ and APP

Abnormalities in early brain development linked to autism may predispose to seizures in early age. These include cerebral cortex dysplasia detected in 50% of subjects with idiopathic autism, and heterotopias in the alveus, CA4, and dentate gyrus and dysplasia in the dentate gyrus detected in 89% of dup(15) autism cases but in only 10% of idiopathic autism cases (Wegiel et al., 2012b). Similar data have been published in another study (Stoner et al., 2014). The higher prevalence of developmental neuropathologic abnormalities and malformations in the dup(15) autism versus idiopathic autism may contribute to the high risk of early onset of seizures and sudden death in the former group (Wegiel et al., 2012b). Furthermore, focal patches of abnormal laminar cytoarchitecture and cortical disorganization of neurons, but not glia, were detected in prefrontal and temporal cortex in more than 90% of children with autism, which indicate dysregulated prenatal neurogenesis and layer formation (Stoner et al., 2014).

There is also a postulated genetic link between autism and epilepsy: altered expression of GABA receptors was found to be a genetic risk factor associated with autism, while the GABA receptor B3 has also been indicated as susceptibility gene for childhood absence epilepsy (Urak et al., 2006).

Clinical and neuropathological data indicate that epilepsy is associated with accumulation of amyloid-β in the brain. Application of the amyloid ligand C^11^-labeled Pittsburgh Compound B and positron emission tomography to study of individuals aged 50 to 60 who had the onset of epilepsy at the age of 5.1 ± 4.5 years revealed an increased brain amyloid load in 22% of individuals, versus 7% in the control group. This increase in amyloid load was particularly significant in prefrontal cortex in apolipoprotein E ε4 allele carriers (Joutsa et al., 2017). A neuropathological study revealed also a higher neuronal Aβ accumulation in individuals with early onset of intractable seizures, and with a high risk of sudden unexpected death in epilepsy in autistic subjects with dup(15) compared to subjects with idiopathic ASD (Wegiel et al., 2012a,b). These studies support the concept of mechanistic and functional links between autism, epilepsy and alterations of APP processing leading to neuronal and astrocytic Aβ accumulation and diffuse plaque formation.

Addressing the question if altered processing of APP and accumulation of amyloid-β in the brain are the cause or effect of epilepsy is of great importance. There is evidence that epileptic seizures may dysregulate formation and removal of Aβ. Neuropathological and biochemical studies of temporal lobe epilepsy revealed in the hippocampus increased phosphorylation of APP, its cleavage product amyloid-β*56—considered neurotoxic, and higher levels of Aβ1–42 (Gourmaud et al., 2020). The changes were associated with increased activities of BACE1 secretase and several kinases: c-Jun N-terminal kinase (JNK) and the protein kinase R-like endoplasmic reticulum kinase (PERK) linked to amyloid generation. These alterations have been proposed to contribute to drug-resistance and to cognitive impairment in epilepsy (Gourmaud et al., 2020).

Dysregulation of APP processing and Aβ deposition induced by seizures were detected also in the experimental model of seizures in rats (von Rüden et al., 2020). A single episode of seizures induced in rats by electrical impulses increased within 3–4 days the levels of Aβ and APP in the hippocampus. These changes were preceded by increased expression of membrane-associated STriatal-Enriched protein tyrosine Phosphatase (STEP61) and a decrease of tyrosine phosphorylation in its substrates N-methyl d-aspartate receptor (NMDAR) subunit GluN2B and extracellular signal regulated kinase 1/2 (ERK1/2) (Jang et al., 2016). The interaction between STEP61 activity and Aβ in regulation of synaptic functions will be further discussed below.

The effects of seizures on altered APP processing and enhancement of Aβ accumulation are accompanied by pro-epileptogenic effects of Aβ itself. Clinical data in humans indicate that Aβ may contribute to the generation of epileptic seizures, as spontaneous seizures have a significantly higher prevalence in Alzheimer’s disease (AD) than in age-matched controls (Amatniek et al., 2006). Experimental animal *in vivo* models also revealed that Aβ in various aggregation conditions—depending on the test model— may trigger or enhance seizure incidents. Unprovoked single epileptic seizure incidents in the transgenic model of Aβ overproduction—the mouse strain that carries human genes *APPswe* and *PS1dE9*—occur in 2/3 of the animals after the onset of amyloid plaque formation, suggesting that epileptic seizures result from Aβ induced increased neurons’ excitability (Minkeviciene et al., 2009). Electrophysiological studies in this model showed that Aβ1–42 or Aβ25–35 protofibrils induced significant membrane depolarization and hyperexcitability of pyramidal cells. Similar effects of oligomeric Aβ as well as overproduced APP on neuronal hyperexcitability have been reported in other AD mouse models (Busche et al., 2012). The pro-epileptogenic effects of Aβ have been described also in the 4-aminopyridine-induced seizure model of male Wistar rats — an injection of Aβ enhanced the number induced seizures and prolonged the time to full recovery (Alcantara-Gonzalez et al., 2019).

The effects of monomeric Aβ are enhanced by oligomerization. Soluble Aβ oligomers have been shown to induce epileptic episode, impair synaptic plasticity, can inhibit long-term potentiation, and enhance long-term depression of excitatory synaptic strength in the hippocampus (Hsieh et al., 2006), through the mechanisms discussed below.

Thus, early developmental alterations of APP processing and Aβ accumulation associated with autism promote the incidence of epileptic episodes. Once they occur, they further enhance the dysregulation of Aβ production and processing, causing dysfunction of neurons—particularly of the GABAergic ones—and leading to increased incidence of seizures, forming a vicious circle in the neuronal networks.

## Traumatic brain injury: Aβ and APP

Self-injurious behaviors are frequently associated with ASD. They have been reported in 30–50% of autistic children (Duerden et al., 2012; Soke et al., 2016), while a recent analysis showed lower figures (Steenfeldt-Kristensen et al., 2020). Self-injurious behaviors can result in traumatic brain injuries of various degrees that lead to chronic inflammation with activation of microglia (review: Mannix and Whalen, 2012).

Traumatic brain injuries are among Alzheimer’s disease risk factors which have been linked to enhanced production of APP, altered APP processing and accumulation of Aβ (review: Ramos-Cejudo et al., 2018). They can affect APP metabolism and Aβ accumulation even in young children. A head trauma leading to sudden death syndrome in children aged up to 3 years leads to accumulation of APP in cerebral white matter (Jensen et al., 2014). Traumatic brain injury in young adults, even a single event, leads to accumulation of APP in damaged axons and rapid formation of amyloid-β plaques within hours after the incident (Johnson et al., 2010) and leaves widespread classical amyloid-β plaques and diffuse deposits that last for decades of life, as well as multiple neurofibrillary tangles in one third of patients (Johnson et al., 2012). On the other hand, a positron-emission tomography (PET) study of living former National Football League players with cognitive and neuropsychiatric symptoms did not reveal amyloid-β levels higher than controls in brain regions affected by injuries but only higher levels of tau (Stern et al., 2019).

A single severe traumatic brain injury in humans affects Aβ levels in CSF. During the first week after incident the levels of Aβ were shown to be two to three times higher than controls, the Aβ1–42/Aβ1–40 ratio increased about fivefold, without changes of APP levels and normalized during subsequent 2 weeks (Raby et al., 1998). Another study of severe brain trauma detected lower CSF Aβ1–42 levels during a week after injury, particularly in patients who died 6 months post-injury, while blood plasma Aβ42 levels were significantly increased (Mondello et al., 2014). Apparently, the location and extent of brain damage, patient age and other factors may strongly affect Aβ processing and formation of amyloid-β deposits after injuries.

A study of a single controlled cortical injury in triple transgenic AD model mice has shown increased levels of both soluble and insoluble Aβ40 and Aβ42 in the injured cortex within 1 day. Aβ accumulated mainly in damaged axons, and the levels of soluble low-and high-molecular-weight Aβ oligomers were significantly increased in the injured cortex during a week after the insult (Washington et al., 2014). In the light of previously mentioned effects of Aβ on neuronal functions, the days of transient accumulation of Aβ may also be the period of increased vulnerability to epileptic episodes. The expression of APP in the neuronal perikarya and in damaged dystrophic neurites increases from day 1 and lasts for 3 months after brain injury tested in a rat model (Itoh et al., 2009).

The effects of repetitive brain injuries are more prominent. Mild injuries—repetitive but not single— increase brain levels of Aβ and its deposition in human-APP transgenic mice Tg2576, as detected 16 weeks after injuries (Uryu et al., 2002). In this model accumulation of Aβ was associated with cognitive impairments and was linked to increased oxidative stress, with increased urine excretion of isoprostanes (Uryu et al., 2002).

These studies demonstrate that traumatic brain injuries, particularly the repetitive ones, lead to increased production of Aβ and its accumulation in the brain. It should be noted that brain injury in autism may have distinct consequences than in the published studies of human subjects and experiments in APP-transgenic mice, as in autism the APP processing is skewed toward α- and γ-secretase cleavage (Sokol et al., 2006; Bailey et al., 2008; Ray et al., 2011), thus, production of Aβ11–40 and Aβ11–42 is expected to be favored over the full-length species.

## The pathophysiological consequences of Aβ accumulation in the brain

Accumulation of Aβ may have distinct consequences depending on the Aβ species, their concentration, level of their aggregation/oligomerization, as well as brain structures, cell types and subcellular structures where it occurs. In autism—idiopathic and dup(15)—we can expect most prominent effects in amygdala, thalamus, Purkinje cells, and in the neocortex — mainly in the GABAergic PVA^+^ neurons (Wegiel et al., 2012a,b; Frackowiak et al., 2020), however, Aβ deposition in other structures, and in other types of neurons, as well as in glia and blood vessel walls may also strongly affect brain functions.

Multiple Aβ species—N-and C-truncated and post-translationally modified have been described in humans (Kummer and Heneka, 2014). It is generally accepted that the morphological features of fibers depend mainly on the C-terminus while the N-terminus affects toxicity, interaction with cell membranes and degradation (Russo et al., 2002). The intraneuronal, cytoplasmic deposits in autism are composed mainly of the N-tr-Aβ species (Wegiel et al., 2012a,b; Frackowiak et al., 2013). The N-truncated Aβ peptides have been previously considered not to be neurotoxic, however, it has been shown that the peptides Aβ9–42 and Aβ17–42 can assemble into ion channel structures, detected by atomic force microscopy, and can function as calcium channels (Jang et al., 2010). Formation of ion channels upon oligomerization of full-length Aβ is enhanced by some posttranslational modifications (Kumar et al., 2016).

Aβ peptides can be subjected to posttranslational modifications: phosphorylation and cyclization of N-terminal glutamate. Aβ phosphorylation can occur on the serine residues 8 and 26, both modifications have been detected in Alzheimer’s disease brains (Kumar et al., 2016; Hu et al., 2017) but not documented in autism.

The N-terminal truncation the Aβ peptide at the amino acid 3 or 11 leaves glutamate at N-terminus, which opens the possibility of glutamate cyclization to pyroglutamate—the process catalyzed by glutaminyl cyclase, mainly in the acidic secretory vesicles (Cynis et al., 2008). Pyroglutamate modified peptides 3–40/42 accumulate in plasma membrane and in the cytoplasm and form oligomers and aggregates resistant to degradation (Russo et al., 2002), however, their accumulation in autism has not been documented. The pyroglutamate modification of Aβ11–40 increases cytotoxicity of the fibrils, even though the fibrils’ structure remains very similar to the unmodified fibrillar Aβ11-40, with two β-strands connected by a short turn (Scheidt et al., 2017). Accumulation of Aβ peptides with pyroglutamate-11 has been described in autism (Frackowiak et al., 2020).

The pyroglutamate-modified species Aβ-3pE and Aβ-11pE promote misfolding and seeding of aggregation of other Aβ species; in the case of Aβ-3pE-42 its 5% fraction in Aβ1–42 solutions causes maximal aggregation of antiparallel β-sheet structures and formation of short fibrils (Galante et al., 2016). This process is associated with increased neurotoxicity resulting from a disrupted cellular calcium homeostasis (Galante et al., 2016). The Aβ peptides accumulated in autism form SDS stable oligomers (Frackowiak et al., 2020), hence, are likely to be involved in formation such ion channel-like structures. Thus, the pyroglutamate modification of Aβ peptides yields peptides of enhanced cytotoxicity, more resistant to degradation, more hydrophobic, and more prone to aggregation (Antonyan et al., 2018). However, polymorphism of Aβ oligomers, dependent on multiple Aβ species and oligomerization conditions, affecting reactivity with numerous antibodies (Hatami et al., 2014) do not allow detection of specific oligomers by immunohistochemical methods. It remains to be established if in autism certain specific Aβ species are present in oligomers in particular types of neurons and synapses.

Aβ peptides have been found to affect cellular and neuronal network regulations and functions at several levels: (1) nuclear, by affecting transcription, (2) cytoplasmic by inducing oxidative stress, (3) cell membrane receptors in the perikaryon and synaptic, altering their functions, and (4) neuronal networks, causing their dysregulation.

It is not well established which of the effects of Aβ peptides occur at physiologic concentrations. Most of the effects on cell signaling have been detected in the Alzheimer’s disease context and/or in the APP-transgenic mouse models in which the concentrations of Aβ peptides exceed the physiological ones. Thus, some of the reported Aβ effects may not occur or may have marginal effects on neurons and glia in physiological Aβ concentrations. Since in autism, epilepsy and TBI accumulation of Aβ in the brain exceeds the control levels, various non-physiological effects of Aβ are expected to occur. N-truncation of Aβ detected in autism may alter the effects of the peptides on functions of neurons and glia.

### Aβ peptides in the nucleus

Neuronal nuclei in prefrontal cortex in autism have been shown to accumulate N-tr-Aβ peptides, unmodified and pyroglutamate modified (Frackowiak et al., 2020). Their effects on nuclear functions remain to be defined, yet their nuclear presence in autism suggests their activity as regulators of transcription in some neurons and possibly also in glia. Aβ peptides have been suggested to function as transcription regulators following their detection and a thorough characterization in the nucleus of neuroblastoma cells (Barucker et al., 2014).

Aβ in the nucleus binds a specific, Aβ-interacting DNA domain in the promoters of APP, BACE1, and APOE genes in a sequence-specific manner (Maloney and Lahiri, 2011). Aβ in the nucleus may also repress expression of several genes linked to synaptic plasticity, e.g., Arc, Nur77 and Zif268—the mouse equivalent of human *EGR1* (Dickey et al., 2004). EGR1 is involved in regulation of the expression of glutamic acid decarboxylase (GAD), as the GAD67 promoter region contains an EGR1 binding site; deficient EGR1 mRNA expression correlates with significantly lower levels of GAD67 (Kimoto et al., 2014). Such changes in GAD67 expression leads to lower production of GABA, which may result in increased risk of epileptic episodes. In the nucleus of cortical neurons Aβ1–42 may also affect gene expression through another mechanism, by altering expression of the regulatory short RNA molecules— miRNAs (Dursun et al., 2019).

### Aβ and oxidative stress

Numerous studies showed that aggregated and oligomerized Aβ 1–40/42 can generate reactive oxygen species through binding transitional metals — iron and copper— to His6, His13 and His14, in the N-terminal region of Aβ, whereas the carbonyl of alanine-2 is an oxygen ligand (Drew et al., 2009). N-tr-Aβ species lack this region, but methionine-35 can get oxidized forming a sulfuranyl radical and cause lipid peroxidation (Butterfield and Boyd-Kimball, 2005; Butterfield and Sultana, 2011).

Accumulation of N-tr-Aβ in the cytoplasm of cortical neurons in ASD appears to be almost completely co-localized with lipid oxidation derivatives: malondialdehyde (MDA) and 4-hydroxy-2-nonenal (HNE) and the load of Aβ correlates with their accumulation (Frackowiak et al., 2013). The morphological relationship between N-tr-Aβ deposits and oxidatively modified lipids suggests that N-tr-Aβ accumulation initiates oxidative stress. The presence of lipid peroxidation products in almost all mitochondria, in some autophagic vacuoles and lysosomes, and in all lipofuscin granules (Frackowiak et al., 2013) reflects the sites of formation of oxygen free radicals and their transport and suggests a deep dysfunction of the affected PVA^+^GABAergic neurons and other cells with high loads of N-tr-Aβ. The PVA^+^ interneurons have been shown to be particularly vulnerable to damage and to damage of perineuronal nets enwrapping these interneurons, as tested in animal models of oxidative stress induced in the brain by genetic alterations or environmental factors (Steullet et al., 2017). Increased oxidative stress was also found to be associated with deposition of Aβ that developed after repeated mild head injuries in Tg2576 transgenic mice (Uryu et al., 2002).

Oxygen free radicals are generally known to modify cytoplasmic proteins. They can also trigger deposition Aβ — documented in brain vascular smooth muscle cells (Frackowiak et al., 2004).

Accumulation of HNE and MDA has itself very significant biological effects. Lipid peroxidation products can bind proteins in an amino acid–specific way; for HNE the target is histidine (particularly when flanked by basic amino acid residues), also Cys, and Lys. Covalent binding of HNE to enzymes frequently causes their quick inactivation, e.g., glyceraldehyde-3-phosphate dehydrogenase (Ishii et al., 2003) and ion transporting ATPases (Crifò et al., 2005a,b). Among proteins particularly susceptible to modifications by HNE are enzymes involved in glycolysis and ribosomal proteins (Roe et al., 2007). Thus, the cells accumulating Aβ and lipid peroxidation products in ASD become energy deficient. Furthermore, increased levels of HNE enhance production and accumulation of Aβ by up-regulating expression of BACE-1, the APP secretase-β, through activation of the c-Jun N-terminal kinases and p38 (Tamagno et al., 2005). HNE may also enhance Aβ aggregation by covalent cross-linking of Aβ at multiple locations through 1,4 conjugate addition and/or Schiff base formation, leading to formation of Aβ protofibrils (Siegel et al., 2007).

Hence, according to our hypothesis the intraneuronal deposition of N-tr-Aβ in autism early in the life causes enhanced formation of oxygen free radicals and lipid peroxidation products, which lead to a further increase in formation of Aβ in a self-enhancing vicious circle contributing to neuron dysfunction in autism (Frackowiak et al., 2013).

### Effects of Aβ on membrane receptors and calcium channels

Formation of oligomers greatly alters the biological effects of Aβ. In numerous Alzheimer’s disease-related studies Aβ oligomers have been shown to cause an influx of Ca^2+^ in cultured neurons and neuroblastoma cells through distinct mechanisms. Aβ oligomers can induce the mechanism of excitotoxicity by releasing glutamate from astrocytes and reducing the activity of excitatory amino acid transporters 1 and 2 (EAAT-1 and EAAT-2), responsible for reuptake of glutamate (Acosta et al., 2017). This leads to an excessive glutamate concentration in the peri-synaptic space and causes activation of *N*-methyl-d-aspartate (NMDA) receptors—the extra-synaptic ionotropic glutamate receptors, leading to a rise in intraneuronal Ca^2+^ concentration (Acosta et al., 2017). The Aβ oligomers can also induce glutamate-independent Ca^2+^ influx through a direct activation of extra-synaptic ionotropic glutamate receptors acting as Ca^2+^ channels— the NMDA receptors (Snyder et al., 2005; Alberdi et al., 2010; Sinnen et al., 2016) and α-amino-3-hydroxy-5-methyl-4-isoxazolepropionic acid (AMPA) receptors (Alberdi et al., 2010). Oligomers also alter the mechanical properties of lipid membranes changing the membrane tension which activates the NMDA and AMPA receptors (Fani et al., 2021). Ca^2+^ influx can be also induced by Aβ oligomers through activation of voltage-dependent Ca^2+^ channels (Quan et al., 2016) the activation of transient receptor potential melastatin 2 (TRPM2), (Ostapchenko et al., 2015) and the activation of transient receptor potential A1 (TRPA1) (Bosson et al., 2017). Aβ oligomers have also been found to induce a glutamate-independent influx of extracellular Ca^2+^ in cultured neuroblastoma cells and primary neurons by interacting directly with lipid membranes and membrane proteins causing destabilization or perforation of the lipid bilayer (Demuro et al., 2005; Sinnen et al., 2016; Fani et al., 2021). Furthermore, oligomerized Aβ1–42 injected into the mouse hippocampus can alter the expression of specific glutamatergic receptors and transporters: increased expression of the NMDA receptor subunit GluN1 and increased or decreased expression of GluN2A in several subregions have been reported (Yeung et al., 2020). The above effects of Aβ on neuronal activation may be the pathophysiological basis of enhanced risk of epileptic seizures as well as numerous autistic behaviors.

It should be noted that these effects have been detected at high concentrations of Aβ oligomers: 1–5 μM or even 10 μM which are relevant to the AD pathophysiology. It is not known if local brain Aβ concentrations in autism can reach those levels, and if oligomerized N-truncated Aβ can exert similar effects, thus the relevance of these mechanisms to ASD remains to be confirmed. However, even low nanomolar concentrations of soluble Aβ oligomers have been shown to increase neuronal excitability (Lei et al., 2016).

### Effects of Aβ on GABAergic signaling

Studies of autism brains revealed the levels of GAD67 and GAD65 decreased to 50% as compared to controls in parietal and cerebellar cortices, dentate, and amygdala (Fatemi et al., 2002; Cellot and Cherubini, 2014). Accumulation of N-tr-Aβ—unmodified and pyroglutamate-11 modified—in the prefrontal cortex in autism is mainly associated with the PVA^+^ GABAergic neurons, and the high load of N-tr-Aβ is correlated with lower levels of GAD67 (Frackowiak et al., 2020). This decrease, as discussed earlier, may be caused by effects of Aβ accumulation on nuclear transcription, or oxidative modification of GAD in the cytoplasm and reduced production of the GABA neuromediator in the affected cells. Production of the GABA neuromediator is catalyzed by two enzyme isoforms: GAD67 located in the perikaryon, regulated by neural activity, and GAD65 located in synapses (Lazarus et al., 2015). In the context of autism development, it should be noted that reduced levels of GAD67 enzyme can be induced during early development by excessive stress, as detected in the rat model of chronic unpredictable stress (Banasr et al., 2017). Prenatal exposure to maternal stress—as associated with development of ASD (review: Kinney et al., 2008; Beversdorf et al., 2018) specifically depresses precursors of PVA^+^ GABAergic interneurons (Uchida et al., 2014). The fast-spiking PVA^+^ interneurons in the medial prefrontal cortex regulate spatial working memory and coordination of the network activity during goal-driven attention processing. The clinical consequences of dysfunction of PVA^+^ GABAergic neurons and their synapses are much wider and include also cognitive deficits in several psychiatric disorders. Deficiency of GAD67 in PVA^+^ interneurons results in increased excitability of pyramidal cells and cortical dysfunction (Lazarus et al., 2015).

It should be noted that in some cases of autism abnormal GABAergic neuronal development and functions may be directly caused by specific genes linked to autism. A recent study (Paulsen et al., 2022) showed that three autism risk genes—SUV420H1, ARID1B, and CHD8 all caused asynchronous development of the cortical GABAergic and excitatory neuronal lineages leading to abnormal circuit activity. The use of organoid models of the human cerebral cortex from distinct donors, and application of single-cell RNA-sequencing analysis and proteomic analysis revealed shared alterations induced by different autism risk genes even though acting through distinct molecular pathways and modified by the individual genomic background.

Genetic studies have also linked the genes coding for the GABA(A) receptor to predisposition to autism. The chromosome 15q11-q13 region containing three GABA receptor subunit genes is an autism candidate region (Shao et al., 2003). Hence, the differences in the accumulation of N-tr-Aβ and its pyroglutamate modified form between idiopathic autism and dupl-15 with autism (Frackowiak et al., 2020) may result from the fact that the duplicated 15q11-13 section of the human chromosome contains a cluster of three GABAA receptor subunit (GABR) genes, GABRB3, GABRA5, and GABRG3.

### Effects of Aβ on synapses and brain network functions

Accumulation of N-tr-Aβ deposits in the GABAergic synapses — exceeding 7%— may be another marker of dysfunction of the GABAergic system in idiopathic and dupl-15 autism (Frackowiak et al., 2020). In normal cultured neurons endogenous Aβ42 binds to a subset of synapses, mainly glutamatergic (Willén et al., 2017) and damaging effects of aggregated Aβ on axon terminals were found to be more prominent in cholinergic and glutamatergic neurons than in GABAergic (Colom et al., 2011). The *in vivo* effects of even low levels of Aβ oligomers can be greatly enhanced by an inflammatory episode associated with an infection during critical stages of embryonic development and early post-natal life, through activation of microglia (Frost et al., 2019). This effect on microglia may be important in the autism pathogenesis, as infections—prenatal and early postnatal—are among the likely triggering factors for development of autism (Smith et al., 2007; Kinney et al., 2008).

Aβ can alter the excitatory - inhibitory balance in autism by disturbing the functions of the glutamatergic neurons which accumulate Aβ. Aβ inhibits the synaptic plasticity in glutamatergic neurons (Lei et al., 2016) and causes enhanced endocytosis of synaptic NMDA and AMPA receptors — regulated by the α-7 nicotinic receptor, protein phosphatase 2B (PP2B) and the tyrosine phosphatase STEP61 (Snyder et al., 2005; Hsieh et al., 2006). These mechanisms of Aβ-induced synaptic dysfunctions have been proposed for Alzheimer disease pathology.

Synaptic functions can be further affected by increased production of sAPP as the result of epilepsy or brain injury (Kreis et al., 2021). sAPP can bind presynaptic GABA type B receptor subunit 1a (GABABR1a) decreasing synaptic GABA release (Rice et al., 2019). The sAPP 17 amino acid sequence that binds GABABR1a inhibits a spontaneous neuronal activity also *in vivo*, showing that GABABR1a functions a synaptic receptor for sAPP (Rice et al., 2019).

Clinical symptoms in autism, particularly the increased prevalence of epilepsy, indicate altered functions of the inhibitory GABAergic system. The PVA^+^ inhibitory interneurons are essential for maintaining the excitatory/inhibitory balance, high-frequency neuronal synchronization, as well as regulating sensory, cognitive processing, and social behavior. Impairment of their functions leads to dysregulation of the brain excitatory–inhibitory homeostasis in autism and to clinically manifested behavioral disorders. However, the consequences of N-tr-Aβ accumulation, even just in the PVA^+^GABAergic neurons may affect numerous brain functions. Inhibitory synapses of the PVA^+^ and SST^+^ GABAergic neurons are regulated by excitatory neurons through different postsynaptic proteins — either the L-type or R-type calcium channels, respectively (Horn and Nicoll, 2018) are regulated through distinct acetycholine receptor modulators (Demars and Morishita, 2014) and have distinct effects on spatial working memory (Kim D. et al., 2016). In the prefrontal and frontal cortex, the PVA^+^ neurons represent a diverse population that consists of basket and chandelier cells that in layer 3 form a circuitry with pyramidal cells. The fast-spiking PVA^+^ interneurons in the medial prefrontal cortex appear to be involved in coordination of the activity in the local network during goal-driven attention processing (Kim H. et al., 2016). Dysfunctions of the PVA^+^GABAergic interneurons in the prefrontal cortex have been linked also to cognitive deficits in schizophrenia (Murray et al., 2015) and other psychiatric disorders (Demars and Morishita, 2014).

The effects of Aβ depend on the brain structure and specific cell population. In the hippocampus Aβ depresses functional activity of GABAergic neurons responsible for the propagation of the theta rhythm without causing an actual cell damage as detected following experimental Aβ injection in rats (Villette et al., 2010). Intraneuronal accumulation of Aβ peptides in the hippocampus leads in animal models to a deep learning deficit, through the mechanism associated with a reduced nuclear translocation of the CREB co-activator, CRTC1, and decreased expression of CRTC1-dependent genes associated with synaptic plasticity: Arc, c-fos, Egr1, and Bdnf (Wilson et al., 2017).

As discussed above, Aβ when assembled into oligomers, can activate neurons by inducing Ca^2+^ influx *via* amyloid channels and NMDA receptors. This influx, however, is largely dependent on formation of synaptic networks and can be prevented by blockers of synaptic transmission as shown in rat hippocampal and cerebellar cultured neurons. Recruitment of distant neurons through synaptic connections spreads excitation and activates more NMDA receptors and voltage-gated Ca^2+^ channels, leading to excitotoxicity. Ca^2+^ response to oligomers has been also detected in non-neuronal cells — dependent on the presence of NMDA receptors (Caballero et al., 2022).

Aβ can interfere with functions of neuronal networks, by inducing synapse weakening, mainly through interaction of Aβ with the tyrosine phosphatase STEP61, mentioned earlier (Zhang et al., 2008). Increased concentrations of Aβ enhance STEP61 activity, increasing the STEP61-mediated dephosphorylation of GluN2B and ERK1/2 leads to NMDAR internalization and ERK1/2 inactivation. Thus, upregulation of STEP61 and downregulation of GluN2B and ERK1/2 phosphorylation mediate compensatory weakening of synaptic strength in response to acute enhancement of hippocampal network activity, whereas delayed decrease in ERK1/2 expression and increase in APP and Aβ expression may contribute to the maintenance of this synaptic weakening (Jang et al., 2016). Epilepsy, even a single electroconvulsive incident, can increase expression of membrane-associated STEP61 which decreases Tyr-phosphorylation of NMDAR subunit GluN2B and ERK1/2 in the hippocampus. Multiple experimental electric impulses lead to a persistent decrease in GluN2B levels and transiently increased production of APP and Aβ. The latter induces an increase in STEP61 activity, making it a part of the molecular mechanism of regulation of STEP61 and of transient weakening of the synaptic strength (Zhang et al., 2010, 2011). These mechanisms may be responsible for epileptic seizures as comorbid conditions in autism (Jang et al., 2016).

Some functions of neuronal networks in autism may be further destabilized by Aβ through its effects on adrenergic and cholinergic regulations. Aβ oligomers have been shown to bind to the α2A-adrenergic receptor, causing enhanced reaction to noradrenaline and activating the GSK-3β kinase that phosphorylates tau (Zhang et al., 2020). The downstream effects observed in mouse models of amyloidosis — tau hyperphosphorylation, neuroinflammation and memory loss — could be stopped by blocking α2A receptor signaling (Zhang et al., 2020). The noradrenergic subcortical nucleus locus coeruleus, is involved in regulating the adaptive deployment and narrowing of attention to task-relevant stimuli. Neurocognitive, neurophysiological, and magnetic resonance imaging studies indicate atypical activity of adrenergic system in autism (rev. Bast et al., 2018), the likely mechanism of frequently observed in autism aberrant attentional functions, reduced reactivity to exogenous stimuli, exaggerated and variable responses to sensory stimuli and atypical fixed focus (review: Bast et al., 2018). The recent study showed in individuals with ASD a lower than in controls activity of locus coeruleus, measured by task-evoked pupil responses, but limited to task-irrelevant stimuli (Granovetter et al., 2020). Altered noradrenergic activity may be the developmental mechanism leading to reduced social interactions and communications.

Aβ can also interact with α-7 nicotinic receptors (Wang et al., 2000; Nagele et al., 2002) — required for Aβ regulated endocytosis of NMDA receptors in cortical neurons in the mechanism dependent on dephosphorylation of the NMDA receptor subunit NR2B at Tyr1472 by protein phosphatase 2B and the tyrosine phosphatase STEP (Snyder et al., 2005).

Accumulation of Aβ may be even involved in generation of abnormal behaviors typical for autism. Among the structures rich in neurons that accumulate Aβ in dup(15) autism is the amygdala (Wegiel et al., 2012a), known to integrate information from cortical and thalamic sensory inputs and to generate fear and anxiety signals. Distinct subtypes of GABAergic neurons in the basolateral and central amygdala contribute to the processing of anxiety information and modulate the anxiety-related behaviors and fear learning (review Babaev et al., 2018). The importance of GABAergic neurons and synapses in the anxiety circuitry is also indicated by successful therapy of anxiety disorders through increasing the GABAergic neurotransmission (review Babaev et al., 2018). The role of amygdala in self-injurious behaviors is also suggested by the ability to reduce such behaviors in ASD using deep brain stimulation in the amygdala region (review Razmkon et al., 2022). The brain structures also associated with self-injurious behaviors are somatosensory cortical and subcortical regions and their supporting white-matter pathways, as shown in the T1-weighted magnetic resonance and diffusion tensor imaging study (Duerden et al., 2014). The authors suggested the alterations in these regions may either reflect a disrupted brain development or remodeling dependent on their increased activity. Previous study demonstrated that the somatosensory region in the parietal lobe is another site of significant accumulation of N-tr-Aβ (Wegiel et al., 2012a). Thus, dysfunction of the neurons loaded with Aβ particularly of the GABAergic neurons in this region and in amygdala may be crucial for development of self-injurious behaviors in autism.

## Conclusion

**GRAPH 1:**
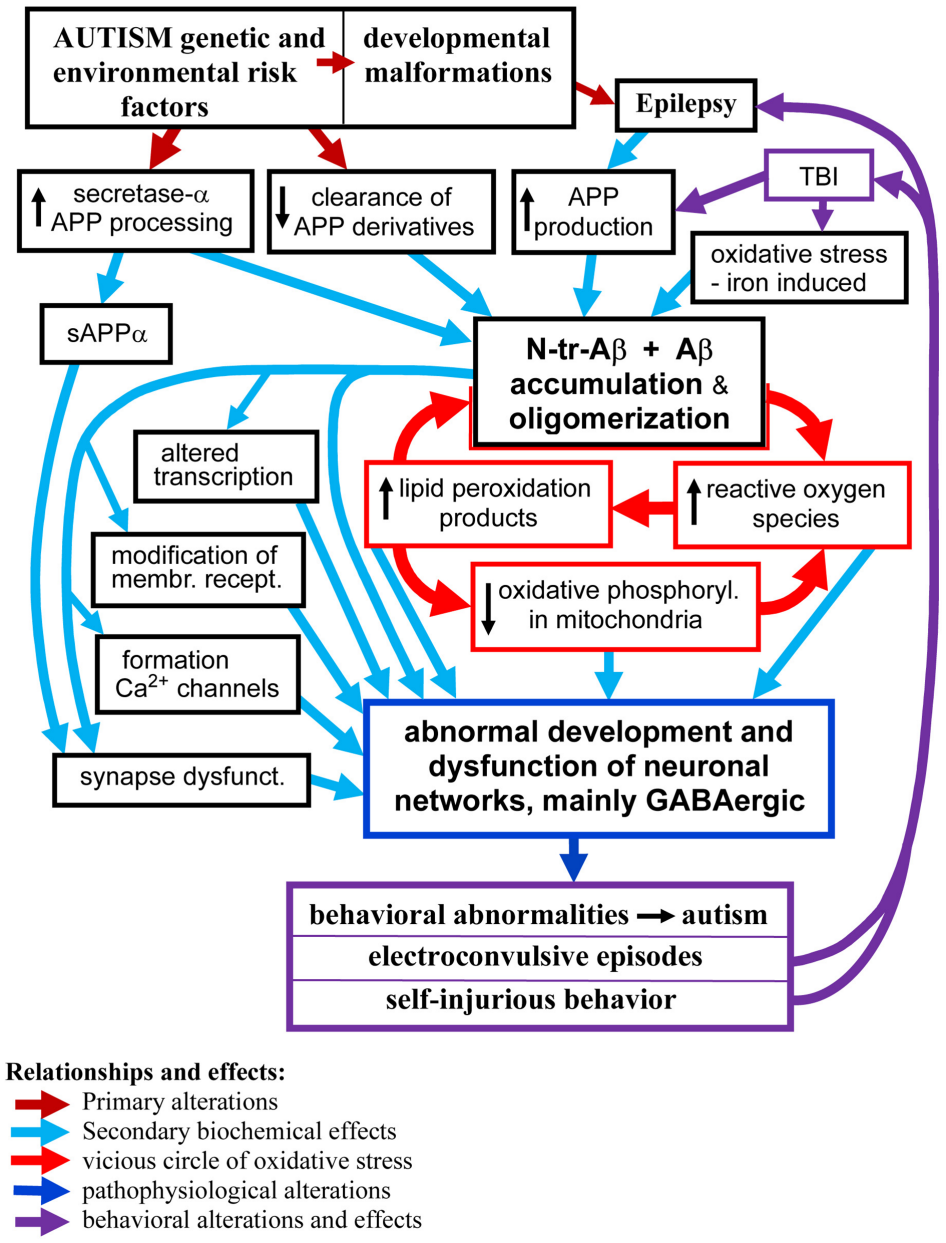
The proposed major relationships between neuronal accumulation of N-tr-Aβ/Aβ and pathomechanisms of neuronal networks’ dysfunction in ASD and co-morbidities. For clarity of the scheme some effects and relationships have been omitted, particularly the pro-epileptogenic effects of Aβ/N-tr-Aβ and their oligomers as well as reactive oxygen species and lipid peroxidation products causing modification of proteins and membrane receptors.

Autism and its co-morbidities all contribute to the enhanced production and accumulation of various species of Aβ peptides which in turn contribute to and enhance dysfunctions of the neuronal networks that manifest as autism symptoms, epilepsy, and self-injurious behaviors. We discuss the biological effects of Aβ peptides in the brain, which depend on the Aβ species, the presence of posttranslational modifications, the local concentration of Aβ, level of aggregation and oligomerization, as well as the brain structures affected, cell types— neurons, glia, vascular cells — and subcellular structures and synapses’ subtypes. Analysis of the published data indicates that Aβ peptides in the brain in ASD, epilepsy, and self-injurious behavior are probably involved in several pathomechanisms, including (1) modulation of transcription—which result in increased APP and Aβ production; (2) induction of oxidative stress, and (3) tilting the excitatory and inhibitory balance in the neuronal networks through (a) activation and alteration of membrane receptors’ signaling; (b) formation of calcium channels causing hyper-activation of neurons and (c) reduction of the inhibitory GABAergic signaling. These changes in the networks’ functions lead to enhanced risk of epilepsy, and likely manifest as clinical symptoms observed in ASD and self-injurious behavior. The proposed relationships between accumulation of Aβ peptides and pathomechanisms of brain dysfunctions in ASD and its co-morbidities, including formation of vicious circles, are shown in the Graph 1.

## Data availability statement

The original contributions presented in the study are included in the article/supplementary material, further inquiries can be directed to the corresponding author.

## Author contributions

JF: concept and the framework of the hypothesis, basic design of the literature data analysis and interpretation, writing the manuscript and design of the graph. BM-K: data analysis and interpretation, search and analysis of the literature and writing the manuscript. All authors contributed to the article and approved the submitted version.

## Funding

Supported with funds from New York State Office for People with Developmental Disabilities.

## Conflict of interest

The authors declare that the research was conducted in the absence of any commercial or financial relationships that could be construed as a potential conflict of interest.

## Publisher’s note

All claims expressed in this article are solely those of the authors and do not necessarily represent those of their affiliated organizations, or those of the publisher, the editors and the reviewers. Any product that may be evaluated in this article, or claim that may be made by its manufacturer, is not guaranteed or endorsed by the publisher.

## Abbreviations

Aβ: amyloid-β peptide
Aβ-pE11: amyloid-β peptide with N-terminal pyroglutamate-11 modification
AMPAR: α-amino-3-hydroxy-5-methyl-4-isoxazolepropionic acid receptor
APP: amyloid-β precursor protein
dup(15): duplication of chromosome 15q11.2-q13 with autism
GAD: glutamic acid decarboxylase
HNE: 4-hydroxy-2-nonenal
5HT3a: the ionotropic serotonin receptor
MDA: malondialdehyde NMDAR *N*-methyl-d-aspartate receptor
N-tr-Aβ: N-terminally truncated Aβ
PVA: parvalbumin
SST: the neuropeptide somatostatin
